# Fatal complications of aortoesophageal fistula caused by pseudoaneurysm following esophageal stent placement: A case report

**DOI:** 10.1097/MD.0000000000041292

**Published:** 2025-01-17

**Authors:** Yiran Liao, Shishi Yu, Neng Shen, Zhongli Liao, Jiong Wang

**Affiliations:** a Phase I Clinical Research Center, Chongqing University Cancer Hospital, Chongqing, China; b Gastroenterology, Chongqing University Cancer Hospital, Chongqing, China.

**Keywords:** aortoesophageal fistula, esophageal stent, hematemesis, pseudoaneurysm, risk assessment

## Abstract

**Rationale:**

Aortoesophageal fistula (AEF) is an exceedingly rare yet critically life-threatening condition, with mortality rates nearing 100% if not addressed promptly. AEF often develops in the context of thoracic aortic aneurysms, esophageal malignancies, or as a complication of foreign body ingestion and prior thoracic aortic surgeries. This study reports an exceptionally severe and clinically rare case of AEF associated with a pseudaneurysm induced by esophageal stenting. By disseminating this case, we aim to heighten awareness of AEF and emphasize the necessity for meticulous decision-making during esophageal stent placement, along with the importance of vigilant postoperative monitoring to enable early intervention.

**Patient concerns:**

A 70-year-old male presented with “food obstruction” and was subsequently diagnosed with esophageal malignancy. To alleviate the obstruction, he underwent esophageal stenting. Fifty-five days postprocedure, he experienced a sudden onset of “chest pain, vomiting of dark red blood, and melena,” prompting hospitalization for “gastrointestinal bleeding.”

**Diagnoses:**

Urgent computed tomography angiography revealed the emergence of a new pseudaneurysm at the lateral aspect of the esophageal stent, with direct communication between the aneurysm and the adjacent esophagus, raising the suspicion of AEF.

**Interventions:**

An urgent multidisciplinary emergency team was convened to execute critical interventions, including endoluminal stenting of the esophagus and thoracic endovascular aortic repair.

**Outcomes:**

The patient suffered a sudden and massive hematemesis, estimated at approximately 3000 mL, leading to his subsequent demise.

**Lessons:**

AEF is a rare cause of upper gastrointestinal bleeding. For patients suspected of AEF, it is imperative to conduct prompt and thorough computed tomography angiography while initiating an emergency surgical alert. The proximity of esophageal stents to the aorta may significantly elevate the risk of AEF; thus, a comprehensive risk assessment should precede stent placement in cases involving tumors adjacent to the aorta. Furthermore, postoperative surveillance is crucial to monitor potential aortic invasion by the tumor or the development of an aneurysm near the esophagus, facilitating timely intervention.

## 1. Introduction

Aortoesophageal fistula (AEF) is a rare yet potentially fatal complication.^[1–4]^ We report the case of a 70-year-old man who developed AEF following the placement of an esophageal stent for a malignant tumor, highlighting the significant risks associated with these procedures. While stent placement is essential for treating dysphagia, it may lead to complications, particularly in patients with adjacent vascular structures like the aorta.^[2,3]^

Diagnosing AEF can be challenging, as it may mimic benign illnesses until serious complications develop.^[1]^ While previous literature has primarily focused on erosive effects, new evidence suggests a multifactorial cause, including mechanical stress from stents.^[1,5–7]^ This case underscores the necessity of thorough preoperative risk assessments and careful postoperative monitoring. By sharing these experiences, we aim to raise awareness of AEF and emphasize the importance of understanding its pathophysiology to improve safety and outcomes for at-risk patients.

## 2. Aim

By sharing this experience, we aim to enhance awareness of AEF and underscore the importance of careful decision-making during esophageal stent placement. Postoperatively, it is crucial to closely monitor for potential aortic invasion by the tumor and the presence of aneurysms in the vicinity of the esophagus, facilitating timely intervention when necessary.

## 3. Case report

**Figure 1.**
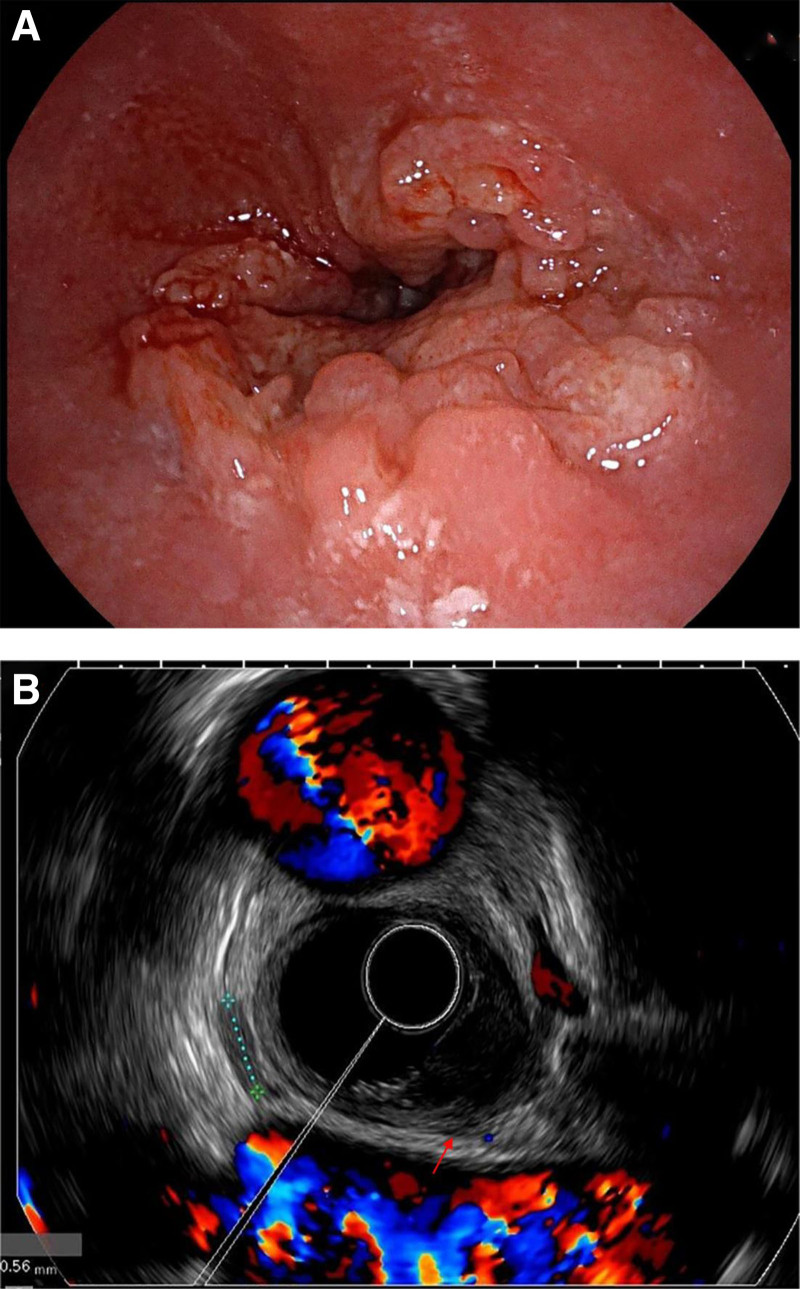
Endoscopic examination of esophageal cancer. (A) Circumferential esophageal lesions with significant esophageal stenosis. (B) Arrows indicate an irregular or shadowless first hyperechoic layer, with a hypoechoic tumor extending into the fifth hyperechoic layer, without invasion of the aorta.

**Figure 2.**
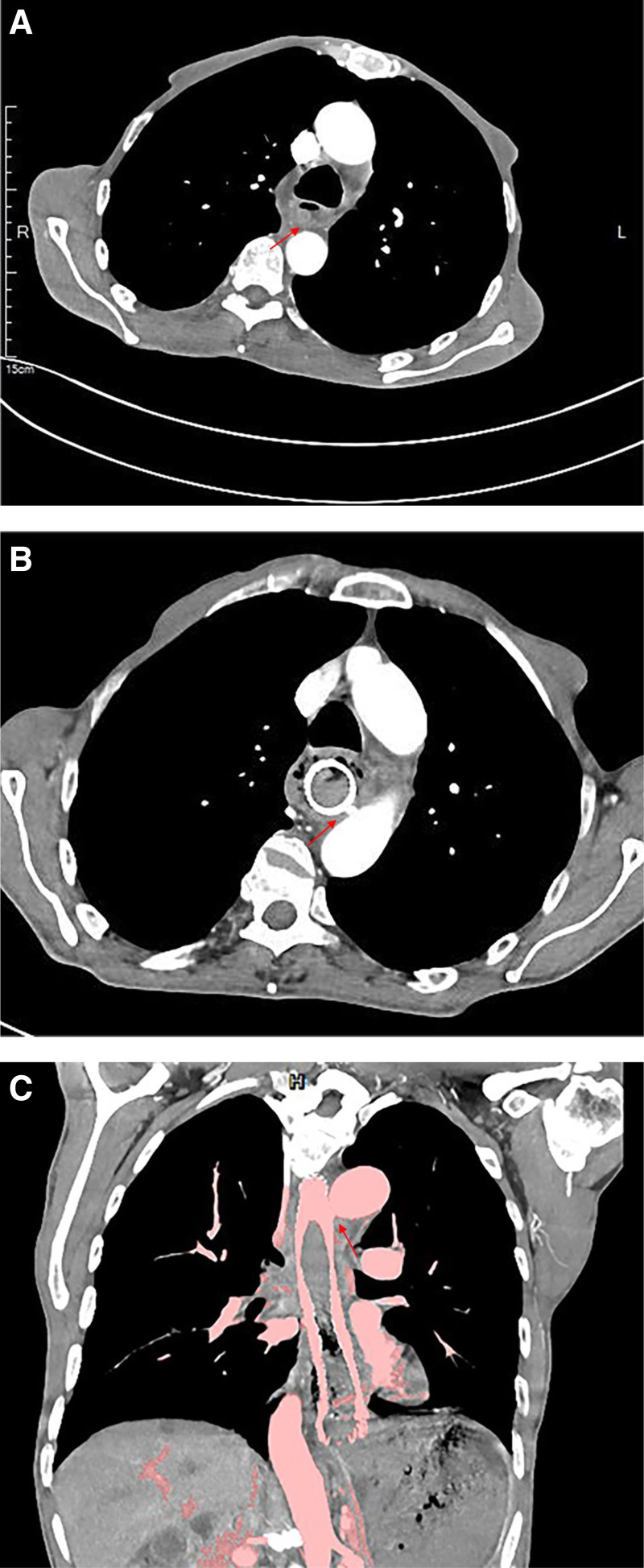
CT images of esophageal stent placement. (A) No false aortic aneurysm was detected at the indicated location prior to esophageal stent placement. (B) and (C) show arrows indicating the presence of a new pseudoaortic aneurysm following esophageal stent insertion. CT = computed tomography.

On December 3, 2023, a 70-year-old man was diagnosed with a malignant esophageal tumor. Endoscopy revealed an ulcerative lesion located 26 to 35 cm from the incisors (Fig. 1A). A pathological examination confirmed a diagnosis of moderately differentiated squamous cell carcinoma. The endoscopic ultrasound indicated infiltration into the muscularis propria and adventitia of the esophagus, with the tumor positioned adjacent to the descending aorta; however, no signs of invasion were observed (Fig. 1B). These findings were confirmed further by computed tomography, which provided additional support for assessing the tumor’s localization features (Fig. 2A). The patient declined all anti-tumor treatment options. To address his dysphagia, a fully covered Polyflex stent (23 × 155 mm) was placed on December 8, 2023. Fifty-five days after the stent placement, the patient experienced sudden chest pain, dark red hematemesis, and melena, resulting in approximately 100 mL of blood loss and 100 g of melena over 11 hours.

**Figure 3.**
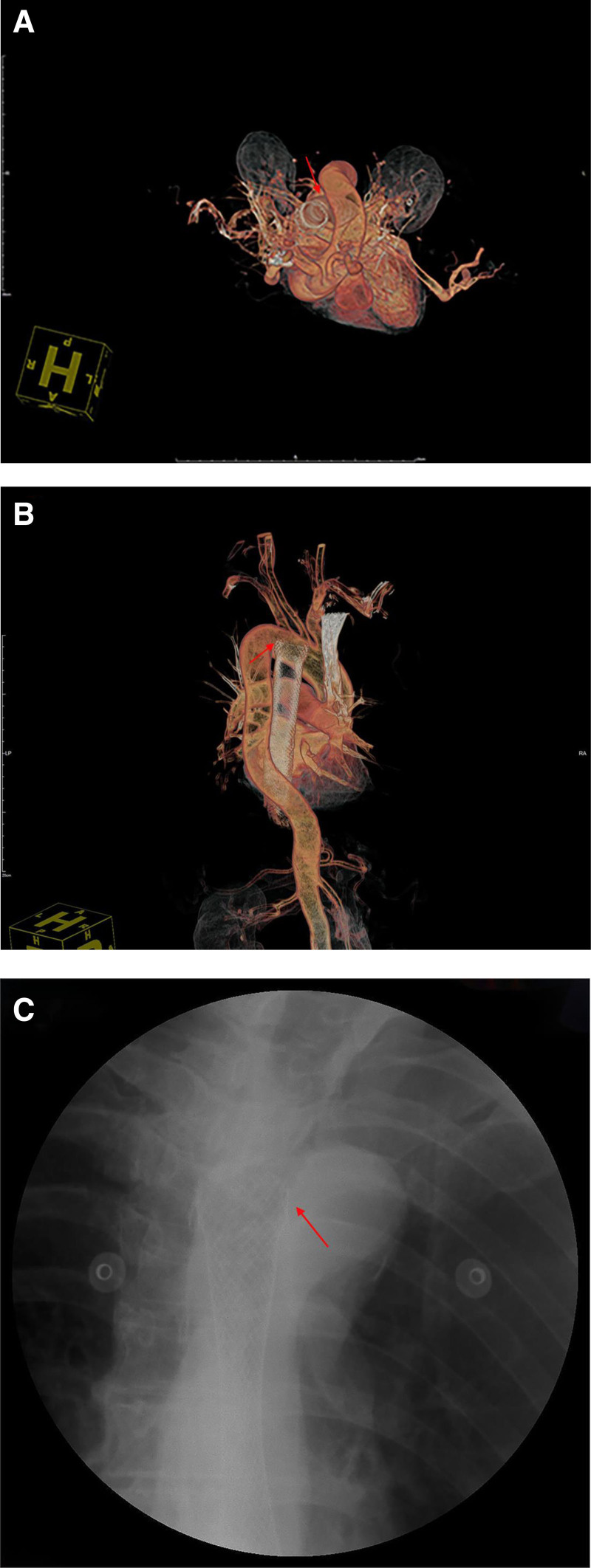
Three-dimensional reconstructed computed tomography image of blood vessels. (A) and (B) show the esophageal stent in contact with the aorta. (C) The esophageal stent remained intact and without any fractures.

Laboratory studies indicated severe anemia, with a hemoglobin level of 56 g/L and an erythrocyte count of 1.74 × 10^12^/L, while cardiac enzyme and electrolyte levels remained within normal limits. The computed tomography angiography subsequently revealed a pseudoaneurysm near the esophageal stent, which suggests the presence of an AEF (Fig. 2B, C). The tumor lesions did not show significant progression compared to the initial diagnosis. The stent remained intact and was snugly positioned against the aorta, with no foreign bodies detected within the aorta (Fig. 3).

An urgent call was issued to a multidisciplinary emergency team, which included a chest oncologist, an interventional radiologist, a gastroenterologist, and a critical care physician. The team was tasked with implementing critical measures, including stent pressure for hemostasis, aortic stent placement, and the administration of broad-spectrum antibiotics. Prior to stent implantation and emergency endoscopy, the patient experienced massive hematemesis estimated at approximately 3000 mL. This significant blood loss resulted in acute hemorrhagic shock, ultimately leading to the patient’s death.

## 4. Discussion

AEF is classified into 2 distinct forms^[1,7–9]^: primary AEF and secondary AEF. Primary AEF typically arises from intrinsic factors such as vascular disease, cancer, or trauma. The causes of primary AEF are diverse and may encompass aortic aneurysms, ruptures, esophageal cancer, perforations, and foreign body ingestion. In contrast, secondary AEF typically develops following endovascular or surgical repair of aortic aneurysms, accounting for approximately 80% of all AEF cases. This form also includes perforations caused by foreign bodies, esophageal malignancies, or complications associated with aortic graft repairs. Notably, secondary AEF frequently occurs as a complication of surgical procedures, particularly following thoracic aortic interventions or stent placements. Understanding the distinction between primary and secondary AEF is essential for comprehending the underlying mechanisms and informing appropriate management strategies.

Atherosclerotic aortic aneurysm is the most common cause of primary AEF.^[1]^ We report a rare case of a pseudoaneurysm that developed following esophageal stenting. Notably, the patient had no prior history of atherosclerosis or pseudoaneurysm. After the esophageal stenting, we observed new pseudoaneurysms near the aorta that contributed to the development of AEF. Previous studies^[8,10]^ indicate that after thoracic endovascular aortic repair, the pulsation of the aorta may lead to friction between the distal end of the stent and the aortic wall. This friction can cause damage to the intima, leading to local blood flow turbulence and increased pressure. As a result, blood can penetrate the layers of the aortic wall, resulting in the formation of distal stent pseudoaneurysms and dilation of the distal stent. To date, there are no reports of false aneurysms induced by extravascular esophageal stents.

This finding highlights the potential role of extravascular mechanical stress and vascular friction in the formation of pseudoaneurysms, providing a new perspective on the pathogenesis of AEF after esophageal stenting. Previous literature has primarily focused on the direct erosion of the esophagus by vascular structures and other invasive mechanisms as the primary cause of AEF.^[1–3,7,11,12]^

Our findings suggest an alternative pathway wherein the mechanical interaction between the stent and the aortic wall may contribute to the development of pseudoaneurysms. This finding could prompt a reevaluation of the traditional understanding of the mechanisms underlying AEF. Specifically, the force exerted by an anchored position or a poorly designed stent may exert local pressure on the aortic tissue, triggering vascular changes that ultimately lead to the formation of a false aneurysm.

However, it is important to note that our study has some limitations. The family’s decision to decline an autopsy limited our ability to investigate potential complications that may have contributed to the patient’s condition. Additionally, the rarity of this case presents further challenges to generalizing our findings. We plan to review the CT scans of patients who underwent esophageal stent placement over the past decade. Our objective is to explore the incidence of aortic complications following esophageal stent placement, identify associated risk factors, and develop a clinical prediction model to aid in decision-making regarding esophageal stent procedures.

## 5. Conclusion

It is essential to recognize that AEF is a rare cause of upper gastrointestinal bleeding. For patients suspected of AEF, prompt and prioritized completion of computed tomography angiography is imperative, alongside the initiation of an emergency surgical alert. The close proximity of esophageal stents to the aorta may constitute a significant risk factor for AEF; therefore, a thorough risk assessment should be conducted prior to stent placement in cases involving tumors adjacent to the aorta, accompanied by the establishment of a stringent postoperative monitoring protocol.

## Acknowledgments

Many thanks to the authors for their contributions to this article.

## Author contributions

**Conceptualization:** Yiran Liao, Jiong Wang.

**Writing – original draft:** Yiran Liao, Shishi Yu, Neng Shen, Zhongli Liao, Jiong Wang.

**Writing – review & editing:** Yiran Liao, Shishi Yu, Neng Shen, Zhongli Liao, Jiong Wang.
